# Transcriptome Analysis of Rice Roots in Response to Root-Knot Nematode Infection

**DOI:** 10.3390/ijms21030848

**Published:** 2020-01-28

**Authors:** Yuan Zhou, Di Zhao, Li Shuang, Dongxue Xiao, Yuanhu Xuan, Yuxi Duan, Lijie Chen, Yuanyuan Wang, Xiaoyu Liu, Haiyan Fan, Xiaofeng Zhu

**Affiliations:** 1 College of Plant Protection, Nematology Institute of Northern China, Shenyang Agricultural University, Shenyang 110866, China; zy648794@163.com (Y.Z.); didizhao@163.com (D.Z.); xiaodx820@163.com (D.X.); xuanyuanhu115@syau.edu.cn (Y.X.); duanyx@syau.edu.cn (Y.D.); chenlj-0210@syau.edu.cn (L.C.); wyuanyuan1225@syau.edu.cn (Y.W.); xwlkitty@syau.edu.cn (X.L.); fanhaiyan2017@syau.edu.cn (H.F.); 2 Shaanxi Key Laboratory of Chinese Jujube, College of Life Science, Yan’an University, Yan’an, Shaanxi 716000, China; shuangli@yau.edu.cn

**Keywords:** root-knot nematode, plant defense, rice, *Meloidogyne incognita*

## Abstract

*Meloidogyne incognita* and *Meloidogyne graminicola* are root-knot nematodes (RKNs) infecting rice (*Oryza sativa* L.) roots and severely decreasing yield, whose mechanisms of action remain unclear. We investigated RKN invasion and development in rice roots through RNA-seq transcriptome analysis. The results showed that 952 and 647 genes were differently expressed after 6 (invasion stage) and 18 (development stage) days post inoculation, respectively. Gene annotation showed that the differentially expressed genes were classified into diverse metabolic and stress response categories. Furthermore, phytohormone, transcription factor, redox signaling, and defense response pathways were enriched upon RKN infection. RNA-seq validation using qRT-PCR confirmed that CBL-interacting protein kinase (*CIPK*) genes (*CIPK5*, *8*, *9*, *11*, *14*, *23*, *24*, and *31*) as well as brassinosteroid (BR)-related genes (*OsBAK1*, *OsBRI1*, *D2*, and *D11*) were altered by RKN infection. Analysis of the *CIPK9* mutant and overexpressor indicated that the RKN populations were smaller in *cipk9* and larger in *CIPK9 OX*, while more galls were produced in *CIPK9 OX* plant roots than the in wild-type roots. Significantly fewer numbers of second-stage infective juveniles (J2s) were observed in the plants expressing the BR biosynthesis gene *D2* mutant and the BR receptor *BRI1* activation-tagged mutant (*bri1-D*), and fewer galls were observed in *bri1-D* roots than in wild-type roots. The roots of plants expressing the regulator of ethylene signaling ERS1 (ethylene response sensor 1) mutant contained higher numbers of J2s and developed more galls compared with wild-type roots, suggesting that these signals function in RKN invasion or development. Our findings broaden our understanding of rice responses to RKN invasion and provide useful information for further research on RKN defense mechanisms.

## 1. Introduction

Rice (*Oryza sativa*) is one of the most important cereal crops. It is a major food source globally and thus has potential for improving global food security [1]. However, rice is vulnerable to injury from a range of pathogens and pests, such as those causing rice blast disease, viruses, insect pests, smut-causing pathogens, and root-knot nematodes (RKNs, *Meloidogyne* spp.). Disease occurrence severely reduces the production yield of rice.

RKNs include several species of *Meloidogyne* (e.g., *Meloidogyne graminicola* and *Meloidogyne incognita*) and are important soil pathogens that significantly influence the yield and quality of rice [2]. Among nematodes, RKNs and cyst nematodes (*Heterodera* spp. and *Globodera* spp.) are the most damaging to plants [3,4,5]. Economic losses of over $100 billion per year have been reported globally due to nematode infection [6]. Second-stage infective juveniles (J2) pierce root cell walls with their stylet at the elongation zone, migrate toward the tip, and then to the plant vascular cylinder to ingest cytoplasmic contents [7,8,9]. Plant cells respond to RKN infection by forming galls or root-knots, which are feeding sites for the parasites.

After feeding, nematodes progress through two developmental stages (J3 and J4) before becoming adults. Adult RKN females are pyriform and remain in the root to begin the next round of production, while males become vermiform and leave the root [10,11]. RKNs secrete effector proteins, which influence host functions, such as cell cycle, cell wall properties, metabolism, and defense, and hijack host cell physiology [12].

Plants have evolved chemical and physical defense mechanisms to protect against pathogen invasion and pathogen infection. Plant hormones such as salicylic acid, jasmonate, gibberellin, abscisic acid, brassinosteroids (BR), cytokinins, and auxin are important for basal immunity in rice [13,14,15,16,17,18]. Several previous studies have reported that plant–nematode interactions affect plant hormones and regulate response mechanisms [19,20]. Recently, sources of genetic resistance to RKNs have been found in the form of nematode resistance genes, which were isolated from different crop plants [21,22]. In rice, *Has-1^Og^* was reported to confer resistance to the cyst nematode *Heterodera sacchari* [23].

Transcriptome analysis is a useful tool to determine transcriptional changes in cells under different conditions [24]. Previous research has provided evidence to suggest that plant–nematode interactions affect the expression of genes associated with plant immune response in dicotyledons [25,26]. Nguyễn et al. (2014) demonstrated that some defense genes in rice were downregulated in the early stages of infection. This suggests that rice is a good model system for studying monocotyledon–nematode molecular interactions [27]. Moreover, increased expression of plant immunity genes in RKN-infected rice, which affects susceptibility to blast disease, has been previously tested using high-throughput sequencing [28]. The results demonstrated that RKN infection also affected oxidative stress response and hormone balance [29]. However, RKN resistance to molecular, physiological, and genetic mechanisms in rice remain poorly understood.

In the present study, the transcriptome profile of RKN-infected rice roots was analyzed, and the differentially expressed genes were annotated using Gene Ontology (GO) and Kyoto Encyclopedia of Genes and Genomes (KEGG). Further genetic study using rice mutants suggested that our approach might be an efficient way to isolate genes associated with resistance to RKN. Overall, our findings provide a theoretical basis for studying the mechanisms underlying RKN defense in rice.

## 2. Results

### 2.1. Analysis of RKN Invasion and Development in Rice Roots

**Figure 1:**
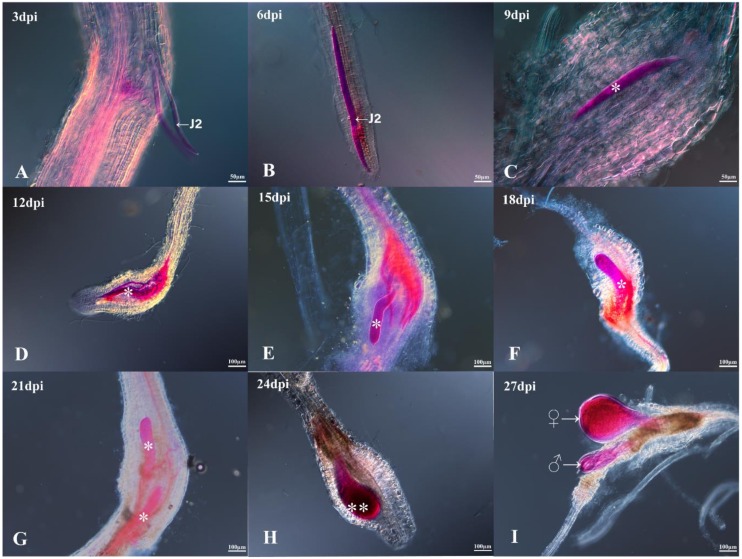
*Meloidogyne incognita* stained with acid fuchsin after infection of Nipponbare rice roots at 3 (**A**), 6 (**B**), 9 (**C**), 12 (**D**), 15 (**E**), 18 (**F**), 21 (**G**), 24 (**H**), and 27 (**I**) days post inoculation (dpi). J2: second-stage infective juveniles; one asterisk: sausage-shaped J2; two asterisks: globose root-knot nematode (RKN); ♀: female adult; ♂: male adult.

Most of the J2 penetrated into roots a few days post inoculation (3 dpi) and then migrated inside the root toward the feeding site. The J2s became sedentary (6 dpi) when they reached the feeding site after passing through the vascular cylinder of the roots. At 9 to 24 dpi, the cells of the feeding sites began to form root-knot galls around expanding giant cells, and the juveniles grew thicker, changing from a sausage-like shape to a globose shape by molting twice. At 27 dpi, the males emerged from the root, while the females continued to thicken and appeared pyriform (Figure 1).

### 2.2. Transcriptome Analysis to Identify Differentially Expressed Genes upon RKN Infection

**Figure 2:**
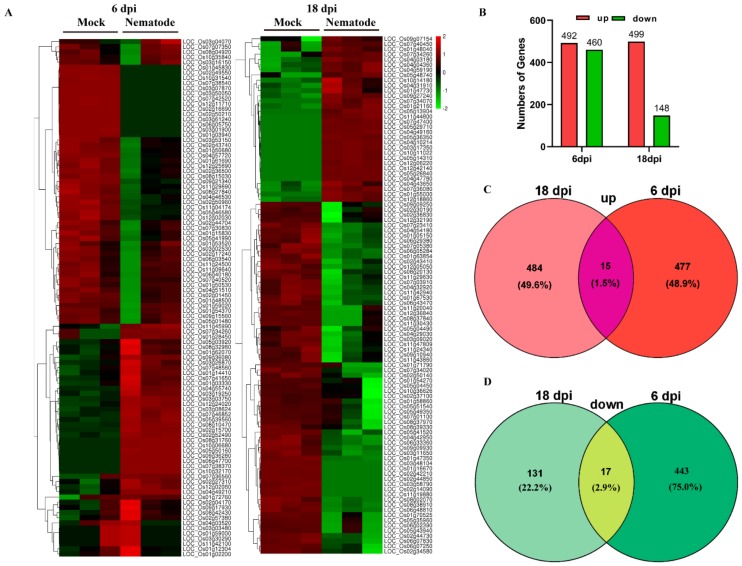
Differentially expressed genes (DEGs) induced by root-knot nematodes at 6 and 18 dpi. (**A**) Cluster heat map showing the level of expression of differential genes; (**B**) Up- and downregulated genes; (**C**) Venn diagrams showing intersection of genes that were upregulated at either 6 or 18 dpi; (**D**) Venn diagrams showing intersection of genes that were downregulated at either 6 or 18 dpi.

Since the J2s were sedentary at 6 dpi and the galls formed between 9 and 24 dpi, RNA-sequencing data from root tissues after 6 and 18 dpi were selected to analyze transcriptome profiles and explore the mechanisms involved in invasion and gall formation. We found 952 and 647 differentially expressed genes (DEGs) after 6 and 18 dpi, respectively (Tables S1 and S2). Among them, 492 and 460 genes were up- and downregulated at 6 dpi, respectively, while 499 and 148 genes were up- and downregulated at 18 dpi, respectively, by RKN infection (Figure 2A,B). In addition, 15 genes were commonly upregulated, and 477 and 484 genes were uniquely upregulated at 6 and 18 dpi, respectively (Figure 2C). Similarly, 443 and 131 downregulated genes were unique to 6 and 18 dpi, respectively (Figure 2D). The 15 commonly upregulated genes included genes encoding actin-depolymerizing factor, myb-like DNA-binding domain-containing protein, lipase, and OsHKT2;1 sodium transporter, while the 17 commonly downregulated genes included genes encoding rhodanese-like domain-containing protein, dihydroflavonol-4-reductase, C3HC4-type domain-containing protein, and RING-H2 finger protein (Table S3).

**Figure 3:**
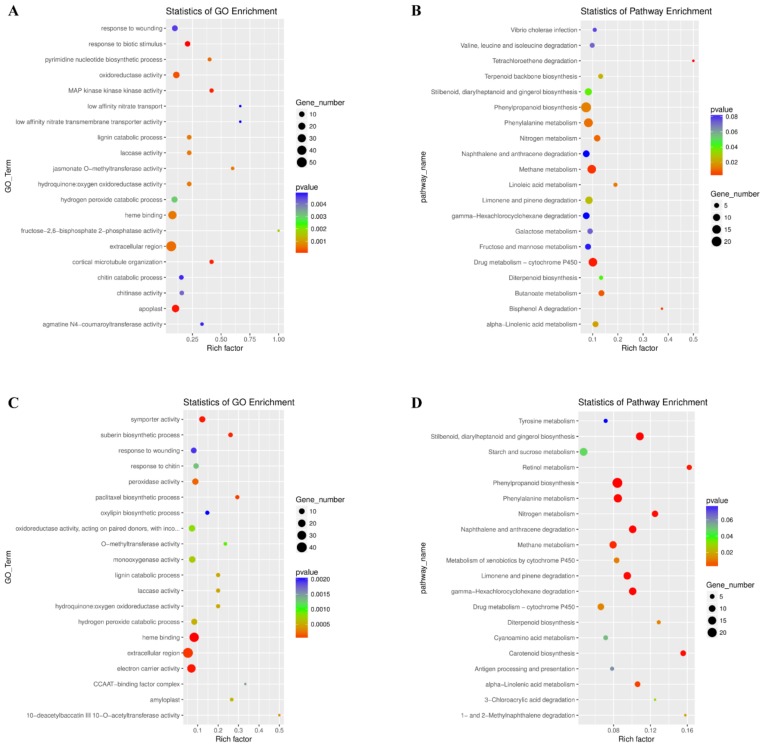
The 20 most enriched Gene Ontology (GO) terms (**A** and **C**) and Kyoto Encyclopedia of Genes and Genomes (KEGG) pathways (**B** and **D**) for Nipponbare rice roots infected by *Meloidogyne incognita* at 6 dpi (**A** and **B**) and 18 dpi (**C** and **D**). “Rich factor” indicates the ratio between the number of DEGs and the number of genes in this pathway. The higher the rich factor, the greater the degree of enrichment.

**Figure 4:**
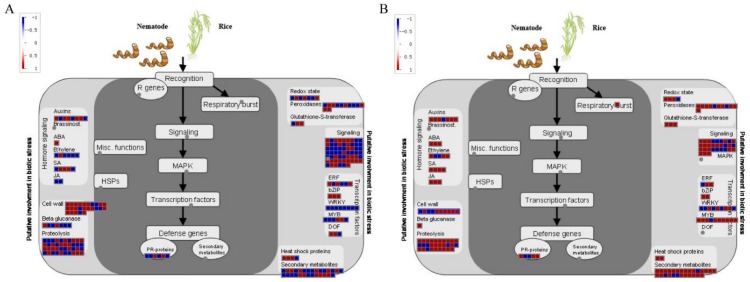
DEG analysis of Nipponbare rice roots infected by *M. incognita* using MapMan (**A**) 6dpi; (**B**) 18dpi.

Furthermore, the DEGs were classified according to GO and KEGG terms. 952 DEGs at 6 dpi were enriched in 20 GO terms, including response to wounding, MAPKKK activity, chitin activity, and chitin catabolic process (Figure 3A). Also, 647 DEGs at 18 dpi were classified by 20 GO terms, including symporter activity, response to wounding, response to chitin, monooxygenase activity, and lignin catabolic activity (Figure 3B). KEGG analysis showed that DEGs at 6 dpi displayed enrichment in diverse metabolic pathways including phenylpropanoid metabolism, phenylalanine metabolism, drug metabolism, and methane metabolism (Figure 3C). In addition, the DEGs at 18 dpi were classified as linked to phenylpropanoid metabolism, phenylalanine metabolism, starch and sucrose metabolism, and methane metabolism (Figure 3D). MapMan analysis was also performed to classify DEGs. This led to the identification of phytohormone- (auxin, BR, abscisic acid, ethylene, salicylic acid, and jasmonate) related, redox signaling, transcription factors (ERF, bZIP, WRKY, MYB, and DOF), and pathogen-related genes (Figure 4).

### 2.3. CIPK Genes in Rice Response to RKN

**Figure 5:**
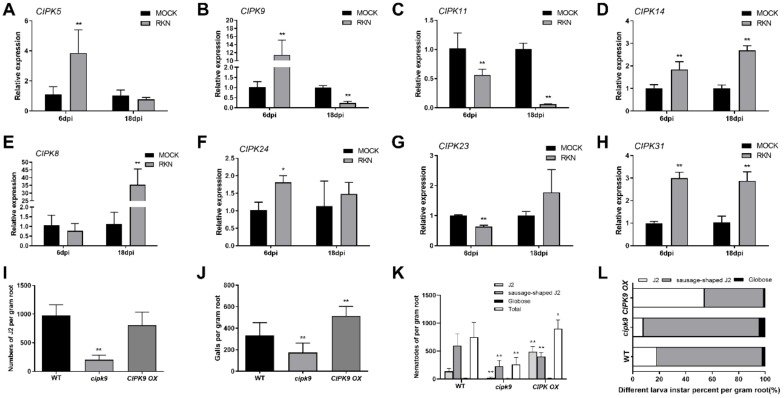
Altered disease resistance of *cipk9* and *CIPK9 OX* plants against RKNs. (**A**–**H**) *CIPK* gene family expression. Error bars indicate the SD between biological repeats (*n* = 3). (**I**) Numbers of second-stage infective juveniles (J2) inside roots on the cipk9 and CIPK9 OX plants at 6 d after RKN inoculation (SD, *n* = 16). (**J**) Numbers of root galls in the cipk9 and CIPK9 OX plants at 18 d after RKN inoculation (SD, *n* = 16). (**K**,**L**) Numbers and percentages of different larval instars on the *cipk9* and *CIPK9 OX* plants at 18 d after RKN inoculation (SD, *n* = 16); * *p* < 0.05, ** *p* < 0.01.

RNA-seq results showed that the expression levels of *CIPK14*(LOC_Os12g02200) and *CIPK23*(LOC_Os07g05620) were altered at 18 dpi (*p* < 0.05, Table S2). In addition, few *CIPK* genes (*CIPK5*(LOC_Os01g10890)*, CIPK8*(LOC_Os01g35184)*, CIPK9*(LOC_Os03g03510)*, CIPK11*(LOC_Os01g60910)*, CIPK24*(LOC_Os06g40370), and *CIPK31*(LOC_Os03g20380)) were listed in the RNA-seq data with altered expressions after RKN-infection, but these differences were not significant (*p* > 0.05, data not shown). To validate the RNA-seq data, qRT-PCR analysis was performed. *CIPK5, 9, 14, 24*, and *31* were significantly induced by RKN infection at 6 dpi compared with the control group, with *CIPK9* exhibiting the highest induction (about 12-fold). Also, *CIPK8, 14*, and *31* were induced at 18 dpi by RKN infection, with *CIPK8* exhibiting the highest induction (about 35-fold). In addition, *CIPK11* and *23* were suppressed at 6 dpi, and *CIPK9* and *11* were suppressed at 18 dpi compared with the control group (Figure 5A–H).

*CIPK* genes were differentially regulated by RKN infection. *CIPK9* was the most highly induced gene at 6 dpi. Therefore, a *cipk9* mutant, a *CIPK9* overexpressor (*CIPK9 OX*), and a wild-type control were used to analyze their responses to RKN infection. After 6 dpi, a fewer J2 populations were observed in *cipk9* mutants than in the wild-type control. However, no significant difference was observed between the wild-type and the *CIPK9 OX* roots (Figure 5I).

The number of galls was determined after 18 days of J2 inoculation, and the results showed that *CIPK9 OX* roots developed more galls than the wild-type and *cipk9* mutant roots. The *cipk9* mutant showed a reduction of galls of almost 32% compared with the wild-type control (Figure 5J). To further evaluate whether *CIPK9* regulates RKN development in roots, the number of J2, sausage-shaped J2, and globose RKN in the roots of each plant were determined.

The results showed that the total number of nematodes was lower in the roots of *cipk9* mutants and higher in those of *CIPK9 OX* plants. The *cipk9* mutant roots contained lower numbers of J2 and sausage-shaped J2, while the *CIPK9 OX* roots contained higher numbers of J2 compared with the wild-type control. *CIPK9 OX* contained a similar number of sausage-shaped J2 to that of the wild-type plants, and the number of globose nematodes was similar in the roots of wild-type, *cipk9*, and *CIPK9 OX* plants (Figure 5K). Furthermore, the results showed that *cipk9* mutants contained a lower percentage of J2, while *CIPK9 OX* contained a higher percentage of J2 than wild-type roots. Sausage-shaped J2 and globose RKNs were present in higher proportion in *cipk9* mutants and in lower proportion in *CIPK9 OX* plants compared with the wild-type control (Figure 5L).

### 2.4. BR Signaling Regulation of RKN Invasion and Development

**Figure 6:**
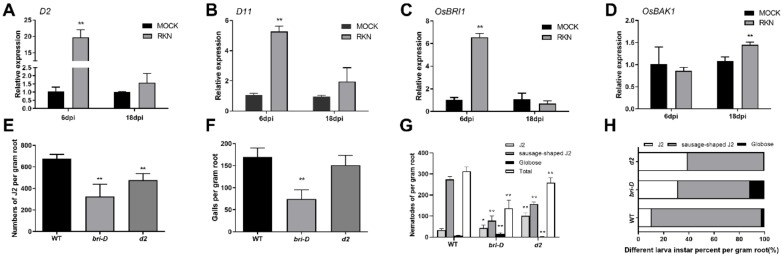
Altered disease resistance of *bri-D* and *d2* plants against RKNs. (**A**–**D**) *D2*, *D11*, *OsBRI1*, and *OsBAK1* expression. Error bars indicate the SD between biological replicates (*n* = 3). (**E**) Numbers of second-stage infective juveniles (J2) inside the roots of *Bir-D* and *d2* mutants at 6 d after RKN inoculation (SD, *n* = 16). (**F**) Numbers of root galls on the *Bir-D* and *d2* mutants at 18 d after RKN inoculation (SD, *n* = 16). (**G**,**H**) Numbers and percentages of different larval instars on the *Bir-D* and *d2* mutant sat 18 d after RKN inoculation (SD, *n* = 16); * *p* < 0.05, ** *p* < 0.01.

Despite *CIPK* genes, a *BR* co-receptor gene (*OsBAK1*(LOC_Os06g16330)) was induced by RKN infection at 18 dpi. To further examine BR signaling and biosynthesis gene response to RKN infection, the expression levels of two biosynthesis genes (*D2*(LOC_Os01g10040) and *D11*(LOC_Os04g39430)), the receptor gene *OsBRI1* (LOC_Os01g52050), and the gene *OsBAK1* were analyzed by qRT-PCR. The results indicated that *D2*, *D11*, and *OsBRI1* were induced at 6 dpi, while *OsBAK1* was induced at 18 dpi compared with wild-type roots (Figure 6A–D). Furthermore, the *d2* mutant, the *OsBRI1* activation tagged mutant *bri1-D* (same as the *OsBRI1* overexpressor) [30], and wild-type control plants (Nipponbare) were used to analyze the effect of BR signaling on RKN invasion and development. After 6 dpi, fewer J2 populations were detected in *d2* and *bri1-D* mutants compared with the wild-type plants (Figure 6E). At 18 dpi, *bri1-D* mutants developed significantly fewer galls compared with the *d2* mutant and wild-type plants (Figure 6F).

To further analyze RKN development, the numbers of J2, sausage-shaped J2, and globose RKN were determined for wild-type, *d2*, and *bri1-D* mutant roots at 18 dpi. The results indicated that the total number of nematodes per gram of roots was lower in *bri1-D* and *d2* mutants than in the wild-type roots. In detail, *bri1-D* and *d2* roots contained more J2 populations but fewer sausage-shaped J2 populations than the wild-type roots. There were more globose RKNs in *bri1-D* roots and fewer globose RKNs in *d2* mutant roots than in the wild-type ones (Figure 6G). Furthermore, the results showed that *d2* and *bri1-D* mutant roots contained significantly more J2 than the wild-type roots. In addition, evidently fewer sausage-shaped J2 were found in *d2* and *bri1-D* roots than in the wild-type ones. However, interestingly, there were more globose RKNs in the *bir1-D* mutant than in the wild-type and *d2* mutant roots. There were fewer globose RKNs in the *d2* mutant than in the wild-type roots (Figure 6H).

### 2.5. Activation of Ethylene Signaling during RKN Invasion and Development

**Figure 7:**
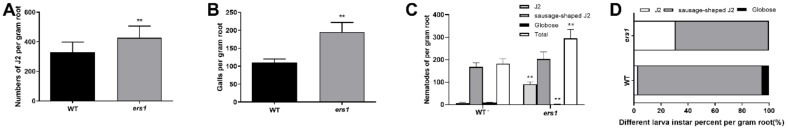
Altered disease resistance of *ers1* plants against RKNs. (**A**) Numbers of J2 in the roots of the *ers1* mutant at 6 d after RKN inoculation (SD, *n* = 16). (**B**) Numbers of root galls in the *ers1* mutant at 18 d after RKN inoculation (SD, *n* = 16). (**C**,**D**) Numbers and percentages of different larval instars in the *ers1* mutant at 18 d after RKN inoculation (SD, *n* = 16); * *p* < 0.05, ** *p* < 0.01.

MapMan analysis revealed that ethylene-related genes and ERFs were enriched. To analyze the role of ethylene signaling in RKN invasion and development, a mutant of ERS1, a negative regulator of ethylene signaling was used [31]. At 6 dpi, J2 populations were significantly more numerous in *ers1* than in wild-type roots (Figure 7A); in addition, *ers1* roots developed more galls after 18 days post J2 inoculation (Figure 7B).

Further examination revealed that the total number of nematodes was higher in the *ers1* mutant than in the wild-type control. Among them, more numerous J2 populations and fewer globose RKN were found in *ers1* compared with wild-type roots. In addition, *ers1* and wild-type roots contained a similar number of sausage-shaped J2 (Figure 7C). Further analysis showed that the proportion of J2 was significantly higher and that of sausage-shaped J2 and globose RKN was significantly lower in *ers1* roots compared with wild-type roots at 18 dpi (Figure 7D).

## 3. Discussion

The *M. incognita*–rice pathosystem can serve as a model to elucidate the interactions between RKNs and the host [27]. To identify different genes expressed in rice roots in both the invasion and the development phases of *M. incognita*, the root tissue was sampled at different time points and visualized by staining with acid fuchsin. Our histological examination demonstrated that most J2-stage RKNs invaded before 3 dpi, and feeding sites were successfully constructed at about 6 dpi. Furthermore, continual development of juveniles feeding on giant cells from 9 to 24 dpi, mature females beginning egg production, and males returning to the rhizosphere from 27 dpi were observed. In the RNA-seq-based transcriptome analysis, 952 and 647 DEGs were identified at 6 and 18 dpi in rice roots. At these two stages, similar numbers of induced genes were observed, but the number of downregulated genes was evidently lower at 18 dpi than at 6 dpi. However, only 15 and 17 commonly up- and downregulated genes, respectively, were found at these two stages, suggesting that the gene response differed in these stages.

DEGs from the two stages were commonly classified as linked to response to wound, lignin catabolic process, laccase activity, and response to chitin. Chitin is an essential component of the nematode egg shells and pharynx, and disturbing chitin synthesis or hydrolysis would lead to the failure of nematode embryos or molting [32]. This suggests that activating chitin catabolism by the induction of chitinase genes, plays a key role in host plant response to RKN invasion and gall formation. Nitrate transport and symporter activity were enriched at 6 and 18 dpi, respectively, implying that nutrient transport might be important in these stages.

At 6 dpi, the MAPK signaling term was identified. Previous research demonstrated that the *MAPK* gene family functions as a cohort during soybean (*Glycine max*) response to *Heterodera glycines* [33]. This suggests a key role for MAPK signaling in plant response to different species of plant parasitic nematodes. KEGG pathway analysis showed that C and N metabolism were enriched at 6 and 18 dpi, suggesting that the host plant actively fine-tunes nutrient metabolism in response to RKNs.

DEG annotation using MapMan suggested enrichment in genes related to phytohormones (auxin, BR, abscisic acid, ethylene, salicylic acid, and jasmonate), redox signaling, transcription factors (ERF, bZIP, WRKY, MYB, and DOF), and pathogens. Previous research demonstrated that in galls at 3 dpi infected with the RKN *M. graminicola*, genes for gibberellin and BR biosynthesis and signaling were activated, while those for salicylic acid and ethylene signaling were repressed [34]. Exogenous application of salicylic acid, jasmonate, or ethylene increased rice response to RKNs, with the strongest effect observed under jasmonate treatment [35]. In addition, ethylene was shown to promote plant defense against RKN by activating jasmonate biosynthesis [35]. Interestingly, the roots of *BRI1* mutants infected with RKNs also showed a 30% reduction in gall numbers [17]. BR biosynthesis or BR receptor silencing increased the susceptibility of tomato plants to RKN, independent of salicylic acid and jasmonic acid signaling [36]. Furthermore, our recent work showed that BR signaling negatively regulated *Arabidopsis* resistance to RKN [37]. This result suggests that the response to RKNs is related to BR signaling.

To analyze phytohormone and RKN interactions, genes involved in BR signaling and BR biosynthesis were examined. The data indicated that BR signaling activation (*bri1-D*) or biosynthesis deficiency (*d2*) inhibited J2 *M. incognita* invasion of rice roots, suggesting the presence of diverse BR functions responding to different species of RKN. In addition, *bri1-D*, in which BR signaling was activated, developed fewer galls in the roots. There were fewer J2 and sausage-shaped J2 RKNs in *d2* and *bri1-D* mutants than in the wild-type roots, while more and fewer globose RKNs were observed in *bri1-D* and *d2* roots, respectively, than in the wild-type ones, suggesting that BR signaling also regulates RKN development in rice roots. In parallel, the expression of *D2*, *D11*, *OsBRI1*, and *OsBAK1* was induced during RKN infection, implying a close connection between BR action and RKN invasion and development in rice roots. Ethylene signaling is known to positively regulate plant response to RKN invasion. In our analysis, *ers1* mutants, in which ethylene signaling was activated, significantly promoted the invasion of J2 RKNs and the formation of galls, suggesting that activation of ethylene signaling inhibits rice root defense against *M. incognita*.

Calcium signaling plays a key role in plant response to environmental change. Previously, calcium/calmodulin-mediated defense signaling was reported to be a key mechanism underlying soybean cyst nematode resistance in wild *Glycine soja* [38]. Furthermore, calcium sensor Cbl10 together with its interacting partner *CIPK6* regulate plant immunity in tomato plants [39]. In the RNA-seq data, we observed a significantly altered expression level of *CIPK* genes in rice roots. Further genetic study using *CIPK9* mutants and the overexpressor revealed that *CIPK9* negatively regulates rice defense to RKN invasion, as indicated by the fewer RKNs observed in the *cipk9* mutant at 6 and 18 dpi. The *CIPK9* mutation promoted RKN development, as indicated by the higher percentage of sausage-shaped J2 populations, while *CIPK9* overexpression increased the total nematode population in roots and inhibited RKN development. *CIPK9* overexpression in plant roots led to the development of more galls than in wild-type plants.

In the *CIPK9 OX*, *bri1-D*, and *ers1* roots, the number of galls appears to be associated with the total RKN population, as shown by the observation that *CIPK9 OX* and *ers1* contained a higher number of total RKNs and galls than *bri1-D* and the wild-type roots. However, the *cipk9* mutant developed a similar number of galls as the wild-type control, even though *cipk9* mutants contained significantly less numerous total RKN populations. This suggests that gall formation was regulated by diverse factors, and the details of the underlying mechanism require further analysis. Our findings will be important for further research on the defense mechanisms of rice against *M. incognita* invasion. Furthermore, the hormone and calcium signaling regulators identified herein might be useful for developing nematode-resistant breeding strategies.

## 4. Materials and Methods

### 4.1. Nematode Culture

*M. incognita* were maintained on a nematode-susceptible tomato cultivar (L402, Liaoning Horticultural Seedling Co. Ltd.). Eggs were collected as described previously [40], with modifications. Briefly, roots of glasshouse-grown tomatoes were cut into pieces and then shook vigorously for 5 min with 10% commercial bleach. Pieces of root were poured through a 180 μm mesh, and eggs were collected on a 25 μm mesh screen. Eggs were quickly purified by centrifugation in 35% sucrose at 720 g for 10 min in a 100 mL centrifuge tube. The supernatant containing the eggs was subjected to another round of centrifugation for 5 min at 720 g in 10% bleach and then rinsed five times in sterile water. Eggs were hatched at 25 °C, and juveniles were collected after passing through 10 layers of Kimwipes tissues into sterile water.

### 4.2. Plant Growth and Nematode Inoculation Assay

*O. sativa* “Nipponbare”, “Dong jin”, *cipk9*: Ds and *CIPK9* overexpressor revealed that *CIPK9* regulates ammonium-dependent root growth [41]; *bri1-D* (BRI1 activation tagging line) in which BR receptor gene *BRI1* was over expressed [42], *d2* rice dwarf mutant with mutation of the gene for the BR biosynthesis enzyme [43], and *ers1* mutant exhibiteing hyper-sensitive response to ethylene treatment [44] were used. The seeds were germinated for 6 days at 30 °C and transferred to plastic Ray Leach containers (SC7 Stubby, diameter, 3.81 cm; depth, 13.97 cm; Stuewe & Sons Inc. Corvallis, OR) that had been filled with sterile sand and further grown at 25 °C under a 16h light/8h dark photoperiod. The plants were inoculated with 1.5 mL of water containing 2000 freshly hatched J2s of *M. incognita*.

For RKN penetration and growth assays, 60 seeds were planted as described above. Ten-day-old seedlings were inoculated with 2000 J2s per seedling and maintained as described previously. Seedlings were gently removed from the container for staining at 3, 6, 9, 12, 15, 18, 21, 24, and 27 days post inoculation. Roots of seedlings were treated with 10% bleach for 1 min, flushed with water, and boiled for 1 min in acid fuchsin solution (3.5% acid fuchsin in 25% acetic acid). Then, the seedlings were transferred to a de-staining acidified glycerin solution (200 μL of 5 mol·L−1 HCl in 50 mL glycerol) that was heated to about 40 °C (not boiled) [45,46]. The number of nematodes inside the roots was counted using a stereoscope (Nikon SMZ800, Nikon, Tokyo, Japan).

### 4.3. RNA Extraction, Library Preparation, and Sequencing

For transcriptome analysis, the roots of Nipponbare rice were collected at days 6 and 18 post RKN inoculation (dpi). For each time point, three independent biological replicates and a control (plant in the same stage but without RKN inoculation) were used for RNA sequencing. Total RNA was extracted using Trizol reagent (Dingguo Biotechnology Co., Ltd., Beijing, China), following the manufacturer’s protocols. Total RNA quantity and purity were analyzed with the Bioanalyzer 2100 and RNA 6000 Nano LabChip Kit (Agilent, Santa Clara, CA, USA) with RIN number > 7.0, and RNA sequencing was performed at LC Biotech Co., Hangzhou, China.

Approximately 10 µg of total RNA was used to isolate poly-(A) mRNA with poly-T oligo-attached magnetic beads (Invitrogen). Following purification, the mRNA was fragmented into small pieces using divalent cations under elevated temperature. Then. the cleaved RNA fragments were reverse-transcribed to create the final cDNA library in accordance with the protocol for the mRNA-Seq sample preparation kit (Illumina, San Diego, CA, USA). The average insert size for the paired-end libraries was 300 bp (±50 bp). Then, we performed paired-end sequencing on an Illumina HiSeq 2000/2500 (LC Sciences, Houston, TX, USA), following the manufacturer’s protocol. To validate the quality of the raw sequence data, forward and reverse reads were analyzed using fastQC (version 0.10.1). Sequencing saturation and 5′ and 3′ biases of RNA-seq data were assessed using RSeQC (version 2.4). Basic data processing consisted of splicing-aware alignment using Tophat (version 2.0.4). RNA-seq reads were aligned to the rice reference genome (https://phytozome.jgi.doe.gov/pz/portal.html#!info?alias=Org_Osativa) by Bowtie (version 2.0.0, Baltimore, MD, USA). Sequences were assembled using StringTie (version 1.3.0, Baltimore, MD, USA), with Ballgown used for differential expression. The original sequencing volume, effective sequencing volume, Q20, Q30, and GC content were determined (Table S4). The raw data were uploaded to the NCBI Sequence Read Arhcive (SAR) database. The BioProject ID is PRJNA589993, and the SRA ID is SRP230566.

### 4.4. Differential Gene Expression Analysis

The DEGs between the two groups of samples were identified using Cuffdiff [47]. Only the genes with a log2 fold change ≥1 or ≤−1 and a *p*-value ≤ 0.05 were considered to be significant DEGs. DEGs associated with biological process GO terms [48] were determined as follows: Fisher’s exact test was used to classify the enrichment of the GO category, and the false discovery rate was calculated to correct the *p* values. The smaller the false discovery rate, the smaller the error in judging *p* [49]. Enrichment of GO terms among probe sets was found using one-tailed Fisher’s exact test based on 2 × 2 contingency tables, because it provides a measure of the significance of the enrichment as it increases, and Pathway analysis was performed against the Kyoto Encyclopedia of Genes and Genomes. For both 6 dpi and 18 dpi, the differentially modulated transcripts were compared and mapped by MapMan software (Version 3.6.0, Cologne, North Rhine-Westphalia, Germany), providing a metabolic overview of transcriptional changes.

### 4.5. qRT-PCR Analysis

For quantitative real-time PCR (qRT-PCR) analysis, total RNA was extracted from samples using Trizol reagent (Dingguo Biotechnology Co., Ltd., Beijing, China) with three biological replications. The cDNA was synthesized from 1 µg of total RNA using the PrimeScript RT reagent kit (Takara Bio, Tokyo, Japan), according to the manufacturer’s instructions. The primers used for these experiments are listed in Table S5. PCR reactions were set up in 96-well hard-shell PCR plates with 0.4 μM primers, using One Step SYBR PrimeScript RT-PCR kit (Takara Bio) in a 10 μL solution. Reaction conditions were as follows: denaturation at 95 °C for 3 min, 40 cycles at 95 °C for 10 s and at 58 °C for 30 s, heating from 60 °C to 95 °C at a rate of 1 °C per 4 s for melt curve analysis. Finally, relative gene expression levels were normalized to the levels of housekeeping gene ubiquitin and calculated using the 2^−△△Ct^ method [50].

### 4.6. Statistical Analysis

All data were analyzed by ANOVA using SPSS version 22 (IBM, Armonk, NY, USA) for statistical significance. Index and density data complied with normality according to the Kolmogorov–Smirnov test and variance homogeneity as determined with the Kruskal–Wallis test. All data were analyzed using paired *t*-test and Duncan’s multiple comparison test.

## Supplementary Materials

Supplementary materials can be found at https://www.mdpi.com/1422-0067/21/3/848/s1.

## Abbreviations

RKNs	root-knot nematode
BR	brassinosteroid
J2	second-stage infective juveniles
GO	Gene Ontology
KEGG	Kyoto Encyclopedia of Genes and Genomes
dpi	days post inoculation
DEGs	differentially expressed genes
qRT-PCR	quantitative real-time PCR
