# Supplementary material for: Transcriptome Analysis of Rice Roots in Response to Root-Knot Nematode Infection
Source: Int J Mol Sci. 2020 Jan 28;21(3):848. doi: 10.3390/ijms21030848 (PMC7037758; doi:10.3390/ijms21030848)
Supplement: Supplementary file 1 [file ijms-21-00848-s001.zip › supplementary/Table S5.docx]

| **Genes** | Forward primers(5’-3’) | Reverse primers(5’-3’) |
| --- | --- | --- |
| ***OsUbiquitin*** | CGCAAGTACAACCAGGACAA | TGGTTGCTGTGACCACACTT |
| ***D2*** | ATGTGATAACAGAGACGCTGCGGT | TGGTGACCAAGTGGTGAAGGAAGA |
| ***D11*** | AGTGAAGAGGGAGCATGAAGGCAT | ATCTGCAGGGCTGAAATTGTTGGG |
| ***OsBRI1*** | CAGCTACTTGGCTATCTTGAAGCTCAGC | CCATTCTTGTTGAAGGTGTACTCCGTGC |
| ***OsBAK1*** | TTTCCTTGTGCATGCTAG | GCATCTCCATAATTGATG |
| ***CIPK5*** | TTCTGCAACAAGGGCCTAAG’ | TCAGGCTCAGCACTTTGATG’ |
| ***CIPK8*** | GTCAAGCATGGAGGTTGTTG | GTTATAATCGGAGGTGTCTC |
| ***CIPK9*** | TGAGCTTCGTAAAACTGGAG | GCATTGGATAGTTGGAATAC |
| ***CIPK11*** | AAGGATATTGTTTGGGTGTG | TGTTTGTTCTGCGGGGATAG |
| ***CIPK14*** | GAAGGAATGGTGTTCTTCAG | ATCTACTCTTGCGACTGCTG |
| ***CIPK23*** | TGGGCTTTAATGTACAGAAG | TCACATCTTTCAGGCCATTG |
| ***CIPK24*** | TGTTGCTGAGACTATGGGTC | GAACCTGTGGTATTCCAGTG |
| ***CIPK31*** | AGTAGCTCCATCCTTACATG | TGGCAAAAACCACGTTCACG |

**Table S5. Primer sequences used in this study.**
